# Differential efferent projections of the anterior, posteroventral, and posterodorsal subdivisions of the medial amygdala in mice

**DOI:** 10.3389/fnana.2012.00033

**Published:** 2012-08-21

**Authors:** Cecília Pardo-Bellver, Bernardita Cádiz-Moretti, Amparo Novejarque, Fernando Martínez-García, Enrique Lanuza

**Affiliations:** ^1^Facultat de Ciències Biològiques, Laboratory of Functional and Comparative Neuroanatomy, Departament de Biologia Cel·lular, Universitat de ValènciaValència, Spain; ^2^Facultat de Ciències Biològiques, Laboratory of Functional and Comparative Neuroanatomy, Departament de Biologia Funcional i Antropologia Física, Universitat de ValènciaValència, Spain

**Keywords:** vomeronasal amygdala, olfactory amygdala, ventromedial hypothalamus, sexual behavior, defensive behavior, chemical signals

## Abstract

The medial amygdaloid nucleus (Me) is a key structure in the control of sociosexual behavior in mice. It receives direct projections from the main and accessory olfactory bulbs (AOB), as well as an important hormonal input. To better understand its behavioral role, in this work we investigate the structures receiving information from the Me, by analysing the efferent projections from its anterior (MeA), posterodorsal (MePD) and posteroventral (MePV) subdivisions, using anterograde neuronal tracing with biotinylated and tetrametylrhodamine-conjugated dextranamines. The Me is strongly interconnected with the rest of the chemosensory amygdala, but shows only moderate projections to the central nucleus and light projections to the associative nuclei of the basolateral amygdaloid complex. In addition, the MeA originates a strong feedback projection to the deep mitral cell layer of the AOB, whereas the MePV projects to its granule cell layer. The Me (especially the MeA) has also moderate projections to different olfactory structures, including the piriform cortex (Pir). The densest outputs of the Me target the bed nucleus of the stria terminalis (BST) and the hypothalamus. The MeA and MePV project to key structures of the circuit involved in the defensive response against predators (medial posterointermediate BST, anterior hypothalamic area, dorsomedial aspect of the ventromedial hypothalamic nucleus), although less dense projections also innervate reproductive-related nuclei. In contrast, the MePD projects mainly to structures that control reproductive behaviors [medial posteromedial BST, medial preoptic nucleus, and ventrolateral aspect of the ventromedial hypothalamic nucleus], although less dense projections to defensive-related nuclei also exist. These results confirm and extend previous results in other rodents and suggest that the medial amygdala is anatomically and functionally compartmentalized.

## Introduction

The main and accessory olfactory systems provide key sensory inputs to the network of neural structures controlling sociosexual behaviors in rodents (Swann et al., 2009). This network is composed of a number of interconnected nuclei rich in neurons expressing receptors for sexual steroids, including the medial amygdaloid nucleus (Me), the posterior bed nucleus of the stria terminalis (BST), the lateral ventral septum, and the medial preoptic area (MPA) (Newman, 1999). Among these structures, only the Me receives convergent projections from the main and accessory olfactory bulbs (AOB) (Scalia and Winans, 1975; Pro-Sistiaga et al., 2007; Kang et al., 2009, 2011). The efferent connections of the Me have been studied in detail in male rats (Canteras et al., 1995) and male hamsters (Gomez and Newman, 1992; Coolen and Wood, 1998; Maras and Petrulis, 2010a). These studies have revealed that the Me is a heterogeneous structure in which at least three subdivisions can be clearly recognized. The anterior division of the medial amygdaloid nucleus (MeA) is connected with structures implicated in defensive, agonistic as well as in reproductive behaviors. The posterodorsal subdivision (MePD) contains the highest density of androgen and estrogen receptors (Simerly et al., 1990; Cooke, 2006) and is connected mainly with structures implicated in reproductive behaviors (Canteras et al., 1995). And finally, the posteroventral subdivision (MePV) projects preferentially to structures suggested being involved in defensive behaviors (Canteras, 2002). These anatomical subdivisions also fit the expression pattern of genes of the Lhx family of transcription factors. Thus, the MeA expresses mainly Lhx5, the MePD Lhx6, and the MePV Lhx9 (Choi et al., 2005). Recent studies on the developmental origins of the Me cells (García-López et al., 2008; García-Moreno et al., 2010; Bupesh et al., 2011) have shown that the Lhx9-expressing cells of the MePV originate in the ventral pallium, and consequently are glutamatergic projection neurons (Choi et al., 2005). In contrast, the Lhx6 neurons of the MePD are GABAergic cells originated in the caudoventral medial ganglionic eminence (and therefore they are neurons of pallidal nature). Finally, the MeA contains a population of Lhx5-expressing neurons originated in the hypothalamic supraoptoparaventricular domain (Abellán et al., 2010) and also abundant nitrergic cells originated from the commissural preoptic area (Hirata et al., 2009; Bupesh et al., 2011).

The neuroanatomical and developmental data discussed above for the different subdivisions of the Me are consistent with a number of functional studies in several rodent species. In mice and hamsters, the MeA has been shown to be activated by sexual and non-sexual social odors, and also by chemicals derived from heterospecific individuals (Meredith and Westberry, 2004; Samuelsen and Meredith, 2009). The MeA seems to categorize the detected chemical stimuli and then relay sex-related information to the MePD (Petrulis, 2009; Maras and Petrulis, 2010b), which consequently is mainly activated by sexually related chemical signals (hamsters: Fernandez-Fewell and Meredith, 1994; Kollack-Walker and Newman, 1997; mice: Choi et al., 2005; rats: Bressler and Baum, 1996; gerbils: Heeb and Yahr, 1996). Accordingly, electrolytic lesions of the transition between the MeA and the MePD are most effective in abolishing the attraction of female mice for sex-derived chemical signals (DiBenedictis et al., 2012) and similar results have been obtained in male hamsters with lesions that functionally disconnect the MeA and the MePD (Maras and Petrulis, 2010c). On the other hand, the MePV display a strong response when mice are exposed to predator (cat) odors (Choi et al., 2005; Samuelsen and Meredith, 2009), a response that has been also reported in rats exposed to cat odors (Dielenberg et al., 2001).

Surprisingly, the efferent connections of the Me in mice have been only partially examined in a single study, which reports data in males mainly regarding the MePD (Usunoff et al., 2009). Given that mice are widely use in behavioral neuroscience studies, due to the availability of genetically modified animals that allow exploring the molecular basis of behavior, it is of interest to obtain direct anatomical data in this species. In addition, since there are relevant differences in different strains of mice regarding reproductive (Vale et al., 1973, 1974; Burns-Cusato et al., 2004; Dominguez-Salazar et al., 2004) and defensive (Belzung et al., 2001; Yang et al., 2004) behaviors, which are major functions of the Me, in the present study we compare the efferent projections of the medial amygdala of the C57BL/6J and CD1 strains of mice. Since our prior behavioral studies about sexual attraction toward male pheromones were performed in females (see Martínez-García et al., 2009), we decided to study the efferent projections of the Me in female mice. This allows comparing the pattern of efferent projections of the Me of females (our findings) with previous published results, which were mostly obtained in males.

## Materials and methods

### Animals

For these studies, we used 17 adult (more than two months of age) female mice *Mus musculus*, from the C57BL/J6 (*n* = 10) and the CD1 (*n* = 7) strains (Charles River, L'Arbresle Cedex, France) with body weights between 18.1–25.1 g and 37.5–45.1 g respectively. Animals were housed in cages with water and food available *ad libitum*, either in natural conditions or in a 12 h light: dark cycle, at 21–22°C. We treat them according to the EEC guidelines for European Communities Council Directives of 24th November 1986 (86/609/EEC), and experimental procedures were approved by the Committee of Ethics on Animal Experimentation of the University of Valencia.

### Surgery and tracer injections

To study the projections arising from the different divisions of the Me (anterior, posteroventral, and posterodorsal), we performed iontophoretic injections of two different dextranamine conjugates as anterograde tracers. Biotin-conjugated dextranamine (BDA, 10,000 MW, lysine fixable, Invitrogen, Carlsbad, CA, USA) was used diluted at 5% in phosphate buffer (PB) 0.01 M, pH 8.0, and tetrametylrhodamine-conjugated dextranamine (RDA, fluoro-ruby, 10,000 MW, lysine fixable, Molecular Probes, Eugene, OR, USA) was used diluted at 10% in PB 0.01 M, pH 7.6. We delivered the tracers from glass micropipettes (10–50 μm diameter tips) by means of positive current pulses (7on/7off s, 3–5 μA, 10–15 min) using a current generator (Midgard Precision Courrent Source, Stoelting). Two to five minutes after the termination of each injection, the pipette was withdrawn while passing retention current (−0.8 μA).

For surgery, animals were anaesthetized either with intraperitoneal injections of a 3:2 ketamine (75 mg/kg, Merial laboratorios, Barcelona, Spain) and medotomedine (1 mg/kg, Pfizer, Alcobendas, Madrid, Spain) solution, complemented with atropine (Sigma, St. Louis, MO, USA; 0.04 mg/kg, IP) to reduce cardio-respiratory depression (*n* = 6) or by inhalation of isofluorane (1.5%) delivered in oxygen (0.9 L/min) (MSS Isoflurane Vaporizer, Medical Supplies and Services Int'l Ltd, UK) using a mouse anaesthetic mask (*n* = 11). We also injected them butorfanol (Fort Dodge Veterinaria, Girona, Spain; 5 mg/kg, subcutaneous) as analgesic. During surgery, animals were on top of a thermic blanket to maintain their body temperature and eye-drops (Siccafluid, Thea S.A Laboratories, Spain) were applied to prevent eye ulceration. After fixing the mouse head in the stereotaxic apparatus (David Kopf, 963-A, Tujunga CA, USA) we drilled a small hole above the medial amygdala. Following tracer injection, we closed the wound with Histoacryl (1050052, Braun, Tuttlinger, Germany). After surgery, animals anaesthetized with the ketamine-medotomedine solution received then injections of atipamezol (Pfizer, Alcobendas, Madrid, Spain; 1 ml/kg, intramuscular) to revert the medotomedine effects.

In the first nine tracer injections (performed in C57 mice) we observed that labeled fibers in the contralateral hemibrain were absent or negligible (see “Results”), so we decided to perform one injection per hemisphere in the rest of the mice, to minimize the number of animals. Following Paxinos and Franklin (2004) in C57mice we used the following coordinates relative to bregma. For MeA: AP −1.1 mm, L −2.0 mm, and D −5.25 mm. For MePD: AP −1.7 mm, L −2.2 mm, and D −5.2 mm. For MePV: AP −1.94 mm, L −2.1 mm, and D −5.3 mm. Coordenates were adapted to CD1 mice as follows: MeA: AP −1.7 to −1.4 mm, L ±2.1 mm, and D −5.10 to −5.3 mm; MePD: AP −1.9 mm, L ±2.1 mm, and D −5.1 mm; MePV: AP −1.9 mm, L ±2.1 mm, and D −5.1 to −5.48 mm.

### Histology

Six to eight days after the injections, animals were deeply anesthetized with an overdose of sodium pentobarbital (Sigma) (90 mg/kg) and perfused with saline solution (0.9%) followed by 4% paraformaldehyde diluted in PB (0.1 M, pH 7.6). Brains were removed from the skull, postfixed for 4 h in the same fixative and cryoprotected with 30% sucrose solution in PB at 4°C until they sank. Using a freezing microtome we obtained frontal sections (40 μm) through the brain that were collected in four matching series. In some animals, the olfactory bulbs were cut at 30 μm.

For the detection of BDA, we inactivated the endogenous peroxidase with 1% H_2_O_2_ [in 0.05 M Tris buffer saline (TBS) pH 7.6] for 15 min and then incubated the sections for 90 min in ABC complex (Vectastain ABC elite kit, Vector Labs, Burlingame, CA, USA) diluted 1:50 in TBS-Tx (Triton X-100 0.3% in TBS 0.05 M pH 7.6). After rinsing thoroughly with buffer, we developed the peroxidase activity with 0.025% diaminobenzidine in PB (0.1 M, pH 8.0), with 0.01% H_2_O_2_ and 0.1% nickel ammonium sulphate. For the RDA immunohistochemical detection, we inactivated the endogenous peroxidase as previously described. Then, sections were incubated overnight in a specific primary antibody against tetrametylrhodamine raised in rabbit (Molecular Probes, Cat. #A-6397) diluted 1:4000 in TBS-Tx, followed by a standard peroxidase-antiperoxidase (PAP) method (goat anti-rabbit IgG, 1:100, Nordic Immunological Laboratories, Tilburg, The Netherlands; rabbit PAP, 1:800, Nordic Immunological Labs). Peroxidase activity was revealed as described before, but nickel was not used.

The sections were mounted onto gelatinized slides, counterstained with Nissl staining, dehydrated with graded alcohols, cleared with xylene and coverslipped with Entellan (Merck, Darmstadt, Germany). We observed the sections using an Olympus CX41RF-5 microscopy and photographed them using a digital Olympus XC50 camera. We arranged the pictures with Adobe Photoshop 7.0 (AdobeSystems, MountainView, CA, USA) and design the line drawings and their labeling using Adobe Photoshop and Adobe Illustrator (Adobe Systems).

## Results

For the description of the distribution of anterograde labeling resulting from the different injections in the Me, we follow the nomenclature of the atlas of the mouse brain by Paxinos and Franklin (2004). To simplify the description of the intra-amygdaloid projections, the term “olfactory amygdala” is used, following Kevetter and Winans (1981a), for the amygdaloid structures that are direct targets of the main olfactory bulb and the term “vomeronasal amygdala” is used to refer to amygdaloid structures that are direct targets of the AOB (Kevetter and Winans, 1981b). The term “chemosensory amygdala” includes both the olfactory and the vomeronasal amygdala (Gutiérrez-Castellanos et al., 2010).

The projection densities in the different targets of the Me subnuclei (anterior, posterodorsal and posteroventral), are subjectively classified as (see Table 1): very dense, dense, moderate, sparse, and very sparse. We considered very dense the projection through the *stria teminalis*, and very sparse the areas where we could observe only 2–5 labeled fibers.

**Table 1:** **Semiquantitative rating of the density of the anterograde labeling resulting after tracer injections in three subnuclei of the medial amygdaloid nucleus**.

		**MeA**	**MePD**	**MePD/PV**	**MePV**
**AMYGDALA**					
	MeA	Injection	+++	++++	+++
Vomeronasal	MePD	+++	Injection	Injection	++++
	MePV	+++	+++	Injection	Injection
	PMCo	+++	++	++++	++++
	BAOT	+++	+++	(not found)	+++
	AAD/AAV	++	++	++	+/++
	AHi	+++	++	++	++
Olfactory	ACo	++	+++	+++	++
	CxA	+	+	++	+↓
	PLCo	+++	++	+++	+++
	LOT	+	+	+	+↓
Central	CeM	++	++	++	+
	CeL	+	+↓	+	+↓
	CeC	+++	+↓	+	–
	Astr	++	–	–	–
	BSTIA	+++	++	++	++
Basolateral complex	BLA	+↓	+↓	+↓	–
	BLP/BLV	+	+	+	+
	BMA	++	++	+++	++
	BMP	+	+	+	++
	La	+	+	+	+↓
**OLFACTORY SYSTEM/CORTEX**					
AOB (MiA/GrA/GlA)	+++/++/+	–	+/+++/−	+/+++/−
Olfactory system	GrO	+↓	–	–	–
	Pir	+	+	+	+
	DEn/VEn	+	+↓	+	+↓
	AON	+	–	+	+↓
	Tenia tecta	++	–	+	+
Cortex	AI	+	–	+↓	+↓
	PrL	+	–	–	–
	IL	+	–	–	–
	Cl	+	–	–	–
Hippocampal formation	Ent	+	+↓	+↓	+↓
	Hippocampus	+	++	+	++
**BST/SEPTUM/STRIATUM**					
BSTL	BSTLD	++↑	–	+	+↓
	BSTLP	++	+↓	+	+↓
	BSTLV	++	+↓	+	+↓
BSTM	BSTMA	++	++	+++	+++
	BSTMPM	++	++++	++++	++++
	BSTMPI	++++	++	+++	++
	BSTMV	++	++	++	+
	BSTMPL	+++	+++	+++	++
Lateral septal complex	LSI	++↑	+	++	+
	LSV	++	+	++	++
	LSD	+	+↓	+	+
	SHy	++	++	++	+++
	SHi	+	+↓	+	+↓
Medial septum/ Diagonal Band	MS	+	–	+	+↓
	HDB	++↑	–	+	+
	VDB	++	–	+	+
Striato-pallidum	VP	+	+	+	–
	Acb	+	+	+	–
	Tu	++	–	+↓	+↓
	ICj	+	–	+↓	+↓
	SI	++	++	++	++
	IPAC	++	–	+↓	+↓
**HYPOTHALAMUS**					
Preoptic	MPA	++	++	++	+
	MPO	++	+++	++	++
	LPO	+	+	+	+↓
	AVPe	–	++	+	–
Anterior	AHA/AHP	++	++	++	+
	Pa	+	+	+	+↓
	Pe	+	+	+	+↓
	LA	+	+	+	+
	SCh	–	+	+	–
Tuberal	VMHDM	+++	+	+++	++
	VMHVL	++	+++	+++	++
	DM	+	+	+	+↓
	LH	+	+	+	+
	Arc	+	+	+	+
Mammilary	PMD	+	+↓	+	+
	PMV	++	+++	+++	+++
	MM/ML	+	+	+	+
	SuM	+	+	+	+
	PH	+	+	+	–
**THALAMUS**					
	SM	+++	+++	+++	++
	Re	+↑	+	+	+↓
	PV	+	+	+	+
	MD	+	–	–	–
	LHb	+	–	–	–
	PT	+	–	–	–
	ZI	+	–	–	–
	STh	+	+	+	+
	PSTh	+	+	+	–
**BRAINSTEM AND MIDBRAIN**					
	PAG	+	+	+	+
	VTA	+	+	+	+
	Rostral linear raphe nucleus	+↓	–	–	–
	Dorsal raphe nucleus	+↓	–		–
	SN	–	+	+	–

++++, Very dense; +++, Dense; ++, Moderate; +, Scarce; +↓, very sparse.

The injection sites obtained in the medial amygdala are described below. In addition, we obtained one injection located in the substantia innominata (SI) above the MeA, and another one restricted to the optic tract (opt) medial to the MeA (not shown). These injections are used as controls for the specificity of the anterograde labeling resulting from the medial amygdala injections (see below).

### Injections in MeA

#### Injections sites

**Figure 1:**
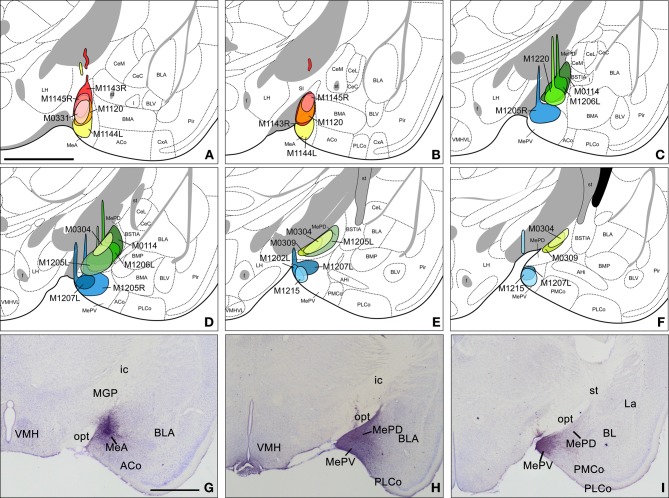
**Injection sites in the anterior, posterodorsal, and posteroventral subdivisions of the medial amygdaloid nucleus. (A–F)** Schematic drawings representing the extent of the tracer injections in the anterior medial amygdaloid nucleus (MeA), the posterodorsal medial amygdaloid nucleus (MePD), and the posteroventral medial amygdaloid nucleus (MePV). MeA injections are represented in warm colors (Figures 1A and B), MePD in green and MePV in blue (Figures 1C–F). Colored areas represent single injections and are identified with the animal code. For those animals with two injections, each one is identified with either an R (right hemisphere) or an L (left hemisphere). **(G–I)** Photomicrographs of nissl-stained sections through the amygdala of the mouse showing representative injections sites. **(G)** Injection site in the MeA of a CD1 mouse. **(H)** Injection site in the MePD of a CD1 mouse. **(I)** Injection site in the MePV of a CD1 mouse. For abbreviations, see list. Scale bar in **A**, valid for **B–I** = 1 mm.

In eight experiments the injection affected the MeA, in five of which the tracer is entirely confined to this subnucleus. Of the restricted injections (Figures 1A,B), two correspond to single injections in the C57BL/6J strain and are used to describe both ipsilateral and contralateral projections of the MeA (Figure 1, injections M0331 and M1120). The remaining three injections, which correspond to CD1 mice, are used to describe the ipsilateral projections. In injections M1143R and M1144L (Figures 1A,B) small tracer deposits appear along the micropipette track, located in the internal capsule (ic), medial globus pallidus (MGP), *SI*, and the opt, and therefore are used only to check the labeling found in restricted injections. In the three additional cases the injections extended caudally and affected also the posterior subdivisions of the Me.

#### Anterograde labeling resulting from injections in the MeA

The injections of neural tracers in the MeA give rise to anterograde labeling in a complex range of cerebral nuclei. Intra-amygdalar axons spread directly from MeA, while fiber labeling coursing outside the amygdala follows two main pathways: the *stria terminalis*, where labeled axons are located in the medial aspect, and the ventral amygdalofugal pathway (*ansa peduncularis*), where axons progress across the *SI*. The output of the MeA is mostly ipsilateral, with scarce fiber labeling present in contralateral nuclei, as described below. Since no differences were observed in the anterograde labeling resulting from experiments in the C57BL/J6 and CD1 strains [except in the olfactory tubercle (Tu) see below], the results obtained in both strains are described together.

**Figure 2:**
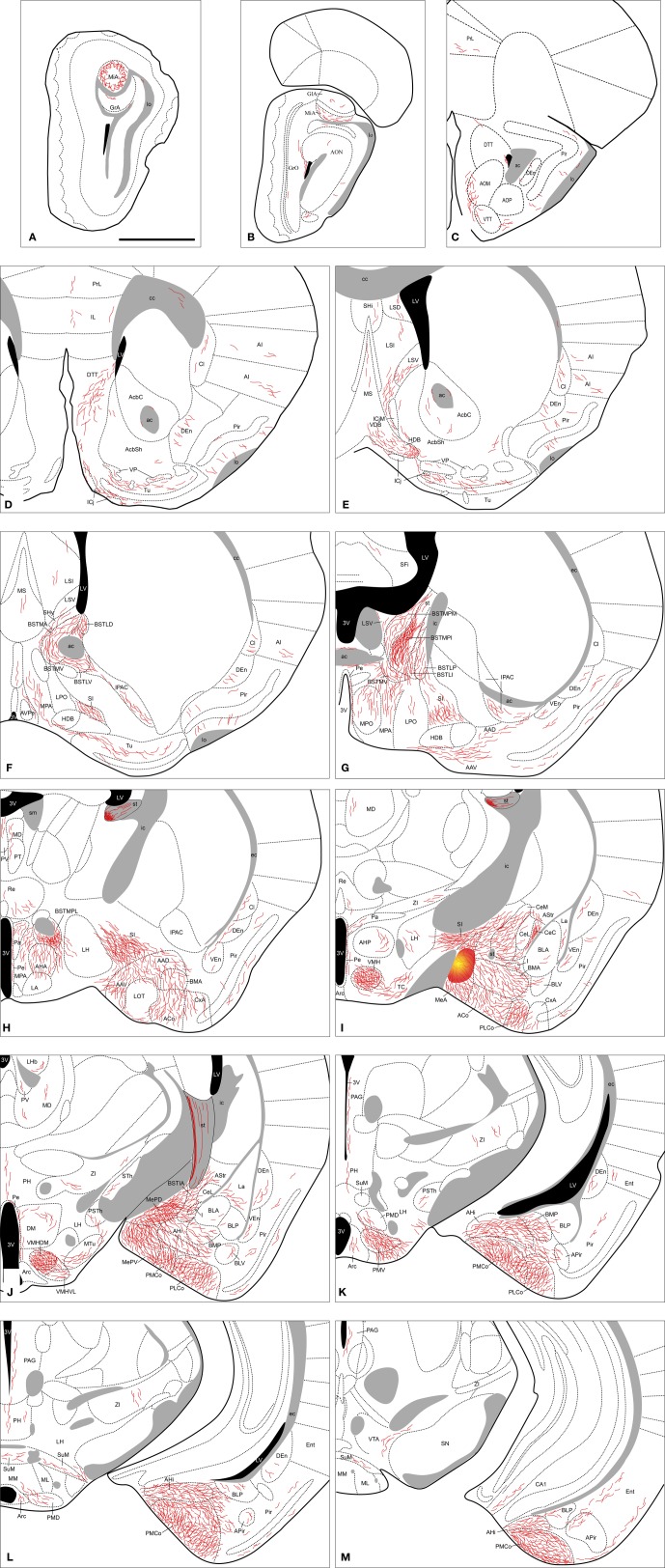
**Semi-schematic drawings of transverse sections through the mouse brain showing the distribution of anterogradely labeled fibers following a tracer injection in the MeA.** The injection site is depicted in panel **(I). A** is rostral, **M** is caudal. The semi-schematic drawings were made based on an injection in a C57BL/J6 mouse. For abbreviations, see list. Scale bar: 1 mm.

**Figure 3:**
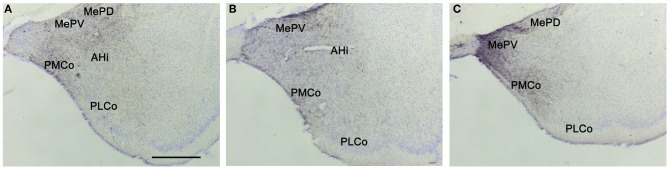
**Anterograde labeling in the amygdala after tracer injections in the different subdivisions of the medial amygdaloid nucleus.** Photomicrographs of transverse sections through the amygdala of CD1 animals receiving tracer injections in the MeA **(A)**, MePD **(B)**, and MePV **(C)**. **(A–C)** Note that in all cases dense anterograde labeling is observed in the vomeronasal amygdala. For abbreviations, see list. Scale bar in **A** (valid for **B** and **C**): 500 μm.

***Amygdala.*** Tracer injections in the MeA give rise to anterogradely labeled fibers that extend to other amygdalar regions (see Table 1). Fiber labeling appears throughout the rest of the Me, with dense terminal fields observed in both MePD and MePV subnuclei (Figure 2J). Dense fiber labeling is observed in the posteromedial (PMCo) and posterolateral (PLCo) cortical amygdaloid nuclei (Figure 3A), amygdalohippocampal area (AHi) (Figures 2I–M) and bed nucleus of the accessory olfactory tract (BAOT) (not shown, see Table 1). Other regions of the chemosensory amygdala display a moderate density of fiber labeling, such as the anterior cortical nucleus (ACo) and the anterior amygdaloid area, mainly in its ventral division (AAV) (Figures 2G,H). Finally, some other chemosensory structures of the amygdala display only sparse anterograde labeling, such as the corticoamygdaloid transition area (CxA), or are mostly devoid of the labeled axons resulting from MeA injections, such as the nucleus of the lateral olfactory tract (LOT) (a small amount of fibers of passage are observed in layer 1 and a few labeled fibers are present in layer 3, Figures 2H,I). In the central nucleus anterogradely labeled fibers give rise to a moderate terminal field in the medial (CeM) and capsular (CeC) parts (somewhat denser at the CeC), and also in the amygdalostriatal transition area, with sparse anterograde labeling observed in the lateral part (CeL) (Figures 2I,J). Dense anterograde labeling appears also in the intra-amygdaloid division of the bed nucleus of the *stria terminalis* (BSTIA) (Figure 2J) and a moderate density in the intercalated mass (I) located between the basolateral and basomedial nuclei (Figures 2I,J). Within the basolateral amygdaloid complex, a moderate density of fiber labeling is present in the anterior basomedial amygdaloid nucleus (BMA) (Figures 2H–K), and only sparse anterograde labeling appears in the lateral (La) and basolateral nuclei (Figures 2I–M).

**Figure 4:**
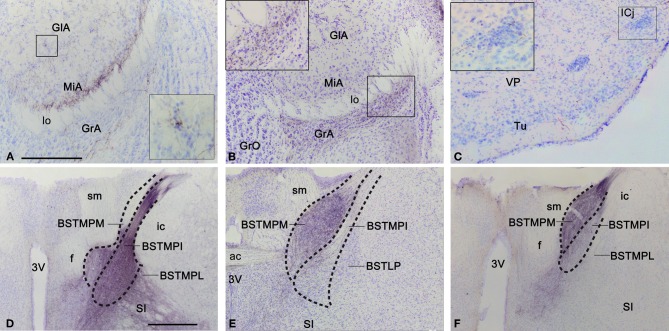
**Anterograde labeling in the accessory olfactory bulb, olfactory tubercle, and bed nucleus of the *stria terminalis* after tracer injections in the medial amygdaloid nucleus. (A)** Nissl-stained transverse section through the accessory olfactory bulb of a CD1 animal with a tracer injection in the MeA, showing the centrifugal projections to the deep aspect of the mitral cell layer. The inset shows a high magnification view of a labeled fiber next to a glomerulus. **(B)** Nissl-stained transverse section through the accessory olfactory bulb of a CD1 animal with a tracer injection in the MePV, showing the centrifugal projections to the granule cell layer. The inset shows a high magnification view of these labeled fibers. **(C)** Photomicrograph of a transverse section through the ventromedial olfactory tubercle of a C57BL/J6 animal with a tracer injection in the MeA. Note that some of the labeled fibers apparently innervate the islands of Calleja. **(D–F)** Photomicrographs of Nissl-stained transverse sections through the bed nucleus of the *stria terminalis* of animals receiving tracer injections in the MeA **(D)**, MePD **(E)**, and MePV **(F)**. Note that in **D** the densest labeling is in the medial posterointermediate division, whereas in **(E)** and **(F)** the densest labeling is in the medial posteromedial division. These photomicrographs correspond to a C57BL/J6 mouse injection **(D)** and CD1 mice injections **(E,F)**. For abbreviations, see list. Scale bar in **(A)** [valid for **(B)** and **(C)**: 250 μm. Scale bar in **D** [valid for **(E)** and **(F)**]: 500 μm.

***Olfactory bulbs and cerebral cortex.*** Fiber labeling arising from the MeA injection extends rostrally through the accessory olfactory tract to end in the AOB, where we observe dense anterograde labeling in the ventral aspect of the mitral cell layer, a moderate density of labeled fibers in the granular layer and a few fibers in the glomerular layer, apparently in the posterior AOB (Figures 2A,B, and 4A). Some labeled axons apposed to the wall of the lateral ventricle run dorsally to the AOB, crossing the granular cell layer of the dorsomedial olfactory bulb (Figure 2B). Fiber labeling from the MeA is also present in the prefrontal cortex, where sparse anterograde labeling is observed in the prelimbic and infralimbic cortices (Figures 2C,D). A moderate amount of fibers is present in the dorsal as well as ventral *tenia tecta* and the piriform cortex (Pir) (with more labeled fibers present in layers 1 and 3) (Figures 2C,D). In addition, a sparse innervation is observed in other cortical regions such as the anterior olfactory nucleus (mainly in the medial and ventral areas), the endopiriform nucleus (Figures 2C–L), the agranular insular cortex and claustrum (Figures 2F–H). Finally, a few axons extend caudally into the entorhinal cortex (Ent) and field CA1 of the ventral hippocampus (Figure 2M).

***Septum, striatum and bed nucleus of the stria terminalis.*** In the septum, at rostral levels there is moderate axonal labeling in the lateral septal complex, which appears denser in its intermediate (LSI) than in its ventral division (LSV), with sparse anterograde labeling appearing also in its dorsal division (LSD) (Figures 2E,F). Some anterogradely labeled axons are also observed in the medial septal nucleus (Figures 2E,F). In addition, moderately dense anterograde labeling is present at the septohypothalamic nucleus (SHy), and a few fibers appear in the septohippocampal nucleus (SHi) (Figures 2E,F). In the nucleus of the diagonal band there is a moderate density of anterograde labeling in the vertical and the horizontal limb, especially in the anterolateral part of the horizontal limb (Figures 2E–G), next to the boundary with the Tu. A number of fibers are also present next to the medial border of the major island of Calleja (Figure 2E).

Within the ventral striatum, a small number of fibers are observed in the nucleus accumbens (Figures 2D,E). There is a moderate labeling in the Tu, with axons mainly surrounding the islands of Calleja (Figures 2D–F and 4C). Only a few fibers enter both the ventromedial and the major islands. This anterograde labeling in the Tu appears denser in the injections performed in the C57BL/6J than in the CD1 animals. In the ventral pallidum, sparse anterograde labeling is observed (Figures 2D,E). In addition, the SI displays a moderate amount of anterograde labeling and fibers of passage belonging to the ventral amygdalofugal pathway (Figures 2F–I). Numerous fibers are also found in the interstitial nucleus of the posterior limb of the anterior commissure (IPAC) (Figures 2F–G).

Fibers arriving to the BST mainly course through the *stria terminalis*, which shows a very dense fiber labeling (Figures 2H–J). The injections in the MeA resulted in dense anterograde labeling in the BST, both in its medial and lateral divisions (see Table 1). A moderately dense terminal field is observed in the lateral BST, especially in the laterodorsal BST (Figures 2F,G). Fiber labeling is also very dense in the posterointermediate medial BST (BSTMPI), dense innervation in the posterolateral medial BST (BSTMPL) and moderate in the anteromedial BST (BSTMA), ventromedial BST (BSTMV), and medial posteromedial BST (BSTMPM) (Figures 2F–H and 4D).

**Figure 5:**
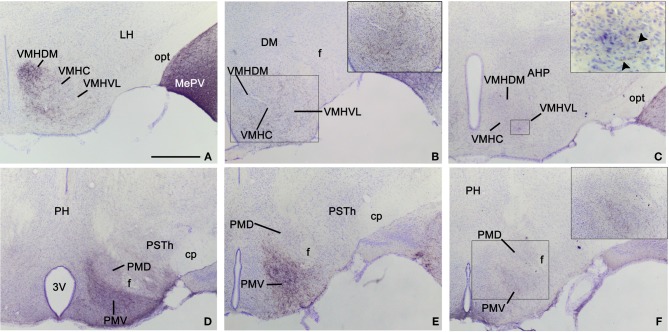
**Anterograde labeling in the hypothalamus following tracer injections in the medial amygdaloid nucleus.** Photomicrographs of transverse sections through the hypothalamus of animals receiving tracer injections in the MeA **(A,D)**, MePD **(B,E)**, and MePV **(C,F). (A–C)** Pattern of anterograde labeling in the ventromedial hypothalamic nucleus (VMH) in CD1 animals. Injections in MeA resulted in dense fiber labeling in the dorsomedial VMH **(A)**, whereas injection in MePD give rise to dense labeling in the ventrolateral VMH **(B)**. The MePV does not appear to innervate preferentially the dorsomedial or ventrolateral subdivision. **(D–F)** Pattern of anterograde labeling in the premammillary hypothalamus. All three subdivisions of the medial amygdaloid nucleus mainly project to the ventral premammillary nucleus, with the densest projections originated by the MeA **(D)** and the MePD **(E)**. Premammillary hypothalamus photomicrographs correspond to a C57BL/J6 mouse injection **(D)** and CD1 mice injections **(E,F)**. For abbreviations, see list. Scale bar in **A** (valid for **B–F**): 500 μm.

***Hypothalamus.*** Following injections in the MeA, abundant anterograde labeling appears in many hypothalamic nuclei (Table 1). At **preoptic** levels, there is a moderate amount of anterograde labeling in the medial preoptic nucleus (MPO) and MPA (Figures 2F,G). A few labeled fibers can also be found in the lateral preoptic area (LPO) (Figure 2G). At **anterior** levels, the anterior hypothalamic area has a moderate labeling (which is less dense in its posterior part) (Figures 2H,I). A low density of labeled fibers is also observed in the lateroanterior hypothalamic nucleus (LA), the paraventricular hypothalamic nucleus (Pa), mainly in its anterior part, and the periventricular hypothalamic nucleus (Pe) (Figures 2F–J). In the **tuberal** region, the ventromedial hypothalamic nucleus shows a heterogeneous fiber labeling. The central (VMHC) and dorsomedial (VMHDM) parts contain dense anterograde labeling, whereas the ventrolateral (VMHVL) part shown only moderate density of fiber labeling (Figures 2I,J and 5A). In addition, a few axons can be observed in the dorsomedial nucleus (DM) and the arcuate nucleus (Arc) (Figures 2J–L). Finally, scarce fiber labeling can be found in the lateral hypothalamic area (LH) (Figures 2H–K). At **mammillary** levels, moderate anterograde labeling is seen in the ventral premammillary nucleus (PMV), with a few fibers present also in its dorsal part (PMD) (Figures 2K,L and 5D). There is also sparse labeling in the supramammillary nucleus (SuM), medial mammillary nucleus (MM), and the posterior hypothalamic area (PH) (Figures 2J–M).

***Thalamus.*** The injections in the MeA do not result in a wide distribution of anterograde labeling in the thalamus. Some axons progress through the *stria medullaris* and apparently reach the paraventricular thalamic nucleus (PV) and the lateral habenula (LHb) (Figures 2H–J). A dense terminal field is observed in the nucleus of the *stria medullaris* (not shown). There is sparse labeling in the nucleus reuniens (Re) (Figures 2H,I), mediodorsal nucleus (MD) and parataenial thalamic nucleus (PT) (Figures 2H–J). Finally, a few labeled axons appear also in the *zona incerta*, subthalamic nucleus (STh) and parasubthalamic nucleus (PSTh) (Figures 2I–K).

**Figure 6:**
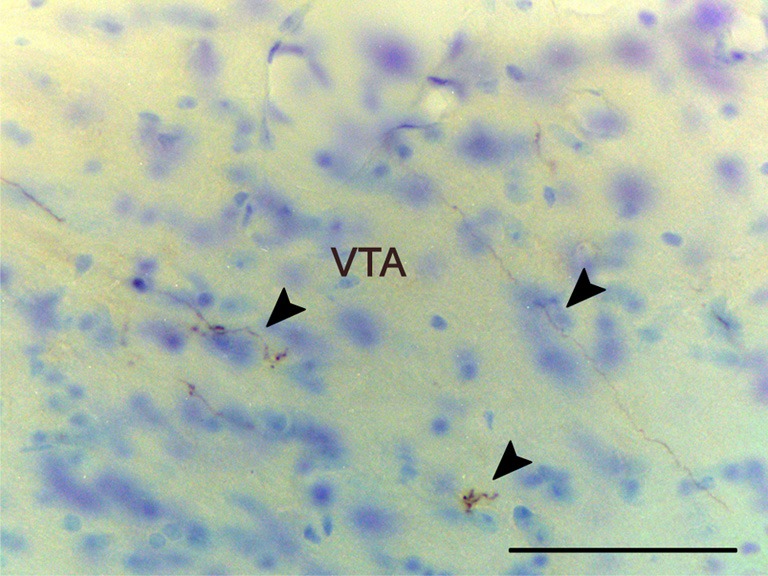
**Anterograde labeling in the ventral tegmental area following a tracer injections in the anterior medial amygdaloid nucleus.** Arrowheads point to scattered labeled fibers. Experiment performed in a CD1 mice. Scale bar: 25 μm.

***Brainstem and midbrain.*** Anterograde labeling is present in the periaqueductal gray (PAG) and the ventral tegmental area (VTA) (Figures 2K–M and 6). Moreover, a few fibers can be seen in the dorsal raphe nucleus and the rostral linear raphe nucleus (not shown).

***Contralateral labeling.*** Although injections in the MeA give rise mainly to ipsilateral labeling, a few axons cross the midline in the anterior commissure, the supraoptic decussation (sox) and the supramammilary decussation. In the contralateral hemisphere, scarce anterograde labeling appears generally in those structures that present a dense ipsilateral projection (with the exception of the contralateral amygdala, which appears devoid of labeling). Thus, a few fibers can be observed in the contralateral *tenia tecta*, Tu, diagonal band, lateral septum, BST, Re, and several parts of the hypothalamus, mainly in MPA, ventromedial hypothalamic nucleus, and PMV.

***Anterograde labeling found in non-restricted injections.*** In the two cases (Figure 1A, injections M1143R and M1144L) where some tracer contamination appears in the ic, MGP, SI, and opt, we observe fibers running through the forceps minor of the corpus callosum (fmi), the external capsule (ec), the basal part of the cerebral peduncle (cp), and sox. As a consequence, very scarce labeling is present in substantia nigra (SN), parafascicular thalamic nucleus, and ventral posterior thalamic nuclei. We observe sparse to moderate fibers in the caudate-putamen (CPu), STh, supraoptic nucleus, and the lateral part of the LHb.

### Injections in MePD

#### Injections sites

The experiments performed resulted in eight injections affecting the MePD. Six of them are confined almost entirely to this nucleus (Figures 1C–F), while two of them affect both the MePD and MePV. Three of the restricted injections correspond to single tracer injections in the C57BL/6J strain and are used to describe the ipsilateral and contralateral projections of the MePD. The remaining restricted injections are done in CD1 mice that received also injections in the contralateral hemisphere, and therefore are used only to corroborate the ipsilateral labeling. In one of the CD1 injections the tracer extended beyond the boundary with the MePV (Figure 1D, injection M1205L). In another case the injection involved the MeA (Figures 1C,D, injection M1206L). In both cases, some of the nuclei or fiber tracts located along the micropipette track present small tracer deposits, including the primary somatosensory cortex, trunk region, the reticular thalamic nucleus (Rt), the ic, the MGP, and the opt (Figures 1C,D, injections M1205L and M1206L).

#### Anterograde labeling resulting from injections in the MePD

As shown for the MeA, the efferents of the posterodorsal region of the Me (MePD) reach a complex range of cerebral nuclei. Axons originated from the MePD follow the same major pathways described for the MeA. However, in this case the majority of the labeled fibers courses through the *stria terminalis*, with less anterogradely labeled axons found in the ventral amygdalofugal pathway. The output of the MePD is mostly ipsilateral, although a few axons appear in contralateral nuclei (described below). As reported above, the anterograde labeling resulting from experiments in the C57BL/J6 and CD1 strains shows no difference.

**Figure 7:**
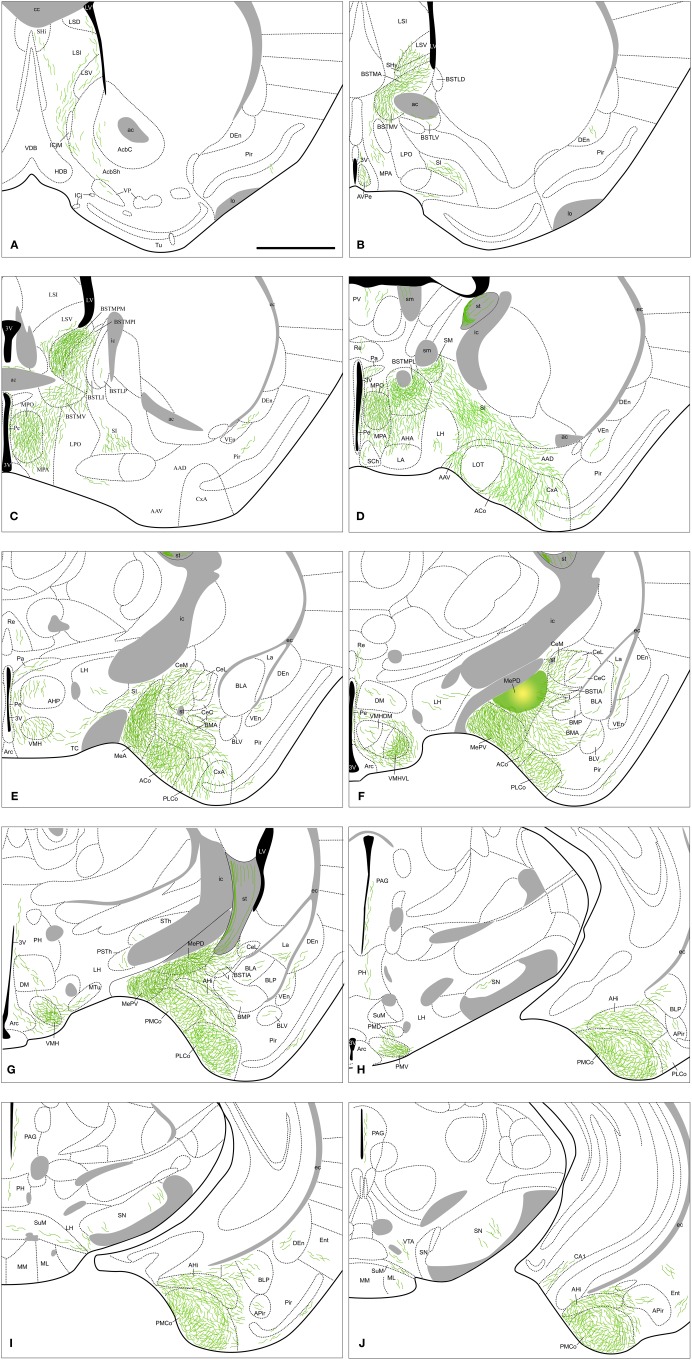
**Summary of the distribution of anterograde labeling following a tracer injection in the MePD, plotted onto semi-schematic drawings of transverse sections through the mouse brain.** The injection site is depicted in panel **(F)**. **A** is rostral, **J** is caudal. The semi-schematic drawings were made based on an injection in a C57BL/J6 mouse. For abbreviations, see list. Scale bar: 1 mm.

***Amygdala.*** From the MePD labeled axons extend to other amygdalar regions (Table 1). There is a very dense anterograde labeling in the rest of the Me (Figures 7E–G). In the cortical amygdaloid nuclei, dense anterograde labeling is present in ACo, and a moderate density of labeled fibers appears in the PLCo and PMCo (Figures 7D–J and 3B). Within the rest of the chemosensory amygdala, the anterior amygdaloid area shows also a moderate density of anterograde labeling, whereas the cortex-amygdala transition zone displays only scarce labeled fibers (Figures 7D,E) and the LOT is mostly devoid of axonal labeling (although some labeled fibers can be observed in layer 1, Figure 7D). In agreement with its proposed association with the vomeronasal amygdala (Swanson and Petrovich, 1998), the AHi also contains a moderate density of labeled fibers (Figures 7G–J) and the BAOT shows dense labeling (Table 1). In the central nucleus, a heterogeneous innervation is found following the injections in the MePD. A moderate density of labeled fibers is observed in the CeM (and also in the BSTIA), whereas sparse fiber labeling appears in the CeL and CeC (and also in the I) (Figures 7E–G). Within the basolateral amygdaloid complex, the basomedial nucleus shows moderately dense axonal labeling in its anterior part (Figures 7E,F). The posterior part of the basomedial nucleus, as well as the basolateral and lateral nuclei, show sparse anterograde labeling (Figures 7F–I).

***Olfactory bulbs and cerebral cortex.*** The anterograde labeling observed in olfactory and cortical structures following injections into the MePD is very scarce. Only a small terminal field appears in the CA1 field of the hippocampus (Figure 7J). Apart from that, sparse labeling is observed in the Pir (with more labeled fibers present in its posterior zone) and endopiriform nucleus (Figures 7A–I), and at caudal telencephalic levels in the Ent (Figures 7I,J).

***Septum, striatum, and bed nucleus of the stria terminalis.*** In the septum, sparse anterograde labeling is present in the dorsal, intermediate, and ventral divisions of the lateral septal complex (Figures 7A–C), with no fiber labeling being observed in the medial septum/diagonal band complex. A moderate innervation is observed in the SHy and a few fibers are present in the SHi (Figures 7A,B). In the ventral striatum, sparse innervation is present in the medial core and shell of the nucleus accumbens (Figure 7A), but no labeling appears in the Tu or the associated islands of Calleja. Anterograde labeling is also very scarce in the ventral pallidum (Figure 7A). Finally, the SI has a moderate labeling as well as fibers of passage coursing through the ventral amygdalofudal pathway (Figures 7B–E).

Regarding the BST, dense anterograde labeling appears in its medial division, especially in the BSTMPM, where it is very dense (Figure 7C, Table 1). In addition, dense anterograde labeling is observed in the BSTMPL and a moderate density of fiber labeling is present in the BSTMA, BSTMV, and BSTMPI (Figures 7B–D and 4E). In contrast, the subnuclei composing the lateral division of the BST show very scarce labeling.

***Hypothalamus.*** In the **preoptic** hypothalamus, dense fiber labeling is observed in the MPO (Figures 7C,D), while the anteroventral periventricular nucleus and the anterior MPA show a moderately dense labeling (a lower number of labeled fibers is observed in the caudal MPA) (Figures 7B–D). A few fibers can also be found in the LPO (Figure 7C). At **anterior** levels, the anterior hypothalamic area has a moderate labeling (especially in its rostral part) (Figures 7D,E). In the rest of the anterior hypothalamus, only a sparse innervation is observed in the LA, suprachiasmatic nucleus, Pa and Pe (Figures 7D,E). In the **tuberal** region, labeled axons resulting after the MePD injections give rise to a dense innervation in the shell of the ventromedial hypothalamic nucleus and in the VMHVL, whereas only a scarce density of labeled fibers is present in the VMHDM and VMHC (Figures 7E–G and 5B). In addition, a few axons can be observed in the DM, Arc, and LH (Figures 7D–G). At **mammillary** levels, the PMV shows dense anterograde labeling, while the PMD shows sparse labeling (Figures 7H and 5E). A few anterogradely labeled fibers are also present in the SuM, MM, and PH (Figures 7G–J).

***Thalamus.*** The injections in the MePD results in scarce anterograde labeling in the thalamus. Only the axons coursing through the *stria medullaris* apparently give rise to dense anterograde labeling in the nucleus of the *stria medullaris* (Figure 7D). In addition, sparse fiber labeling is present in the Re, PV, STh, and the PSTh (Figures 7D–G).

***Brainstem and midbrain.*** In animals with injections in the MePD, some labeled axons run caudally to reach the PAG and to a lesser extent also the VTA (Figures 7H–J). Sparse labeling is also observed in the *SN* (Figures 7H–J), mainly in its reticular part.

***Contralateral labeling.*** The anterograde labeling resulting from the injections in the MePD is mostly ipsilateral, but a few labeled axons are observed to cross the midline in the anterior commissure and the sox. Contralateral labeling is present in the same nuclei that show dense ipsilateral fiber labeling. Thus, sparse anterograde labeling appears in the lateral septum, BST, and the hypothalamus, mainly in MPA, ventromedial hypothalamic nucleus, and PMV.

***Anterograde labeling found in non-restricted injections.*** In the injections M1206L and M1205L, in which tracer contamination occurred in the somatosensory cortex (trunk region), Rt, ic, MGP, and opt, we observe sparse labeling in areas in which no fiber labeling appears following the restricted injections in the MePD. Labeled fibers run through the ec, optic chiasm and the sox, and anterograde labeling is present in the parietal insular cortex, CPu, the ventral posterior thalamic nuclei, posterior thalamic nuclear group (Po), *SN*, and several structures in the visual thalamus.

We observe sparse to moderate fiber labeling in the lateral part of LHb, which also contains labeled axons in the restricted MePD injections.

### Injections in MePV

#### Injections sites

We have obtained four injections confined almost entirely to the MePV, two in the C57BL/6J strain (Figures 1E,F, injections M1202L and M1215) and two in the CD1 strain (Figures 1C–F, injections M1205R and M1207L). In the injections M1205R and M1207L, small tracer deposits are present along the micropipette track, located at the primary somatosensory cortex (trunk region), the cp, the opt, the STh (Figures 1C–F), and caudally the SN, *pars reticulata*. In another case, small tracer deposit are present in the cp, STh, and the pyramidal cell layer of the hippocampus (Figures 1E,F, injection M1215). In addition, to describe the MePV projections we also study the two injections encompassing the MePD and MePV described above (see section “Injections sites”) and an additional one that affected the caudal MePV and the medial aspect of the PMCo. In the case of the MePV the contralateral projections are described based on a C57BL/J6 injection (Figures 1E,F, M1215).

#### Anterograde labeling resulting from injections in the MePV

**Figure 8:**
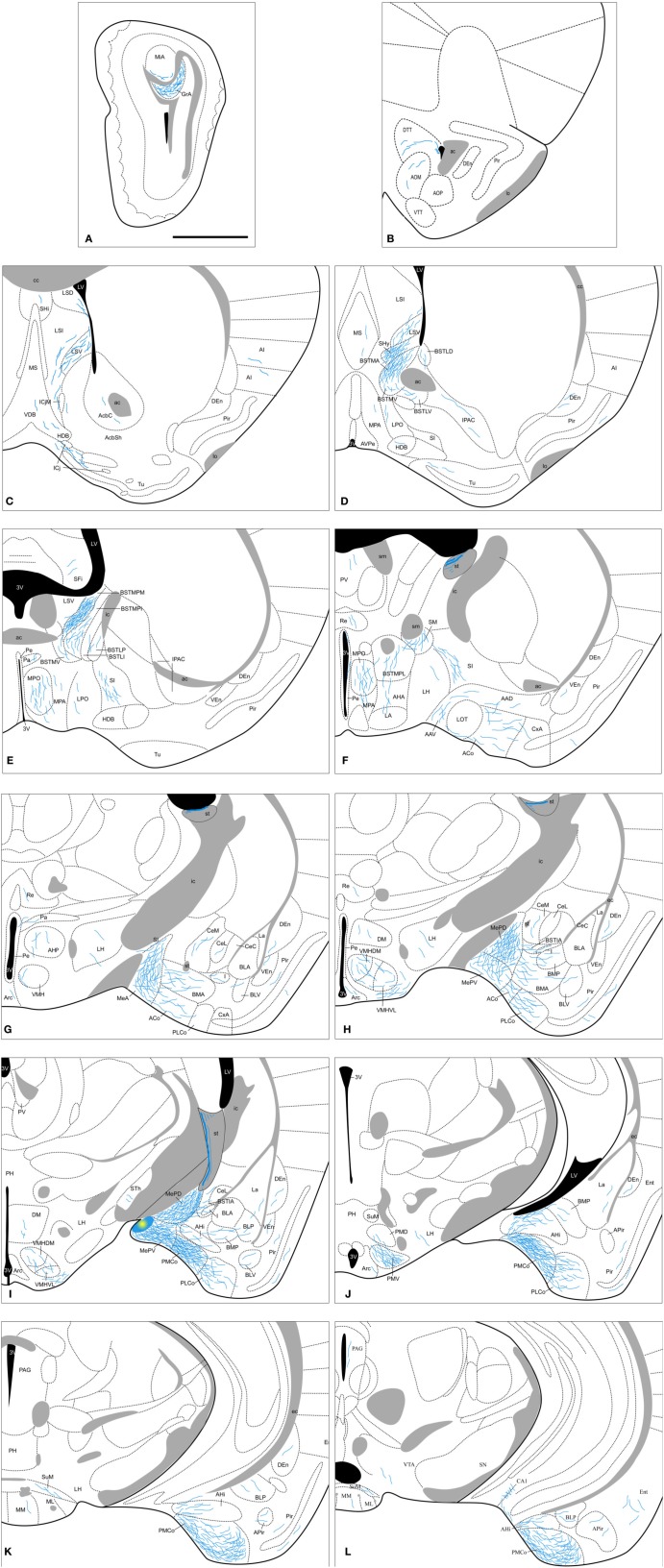
**Semi-schematic drawings of transverse sections through the mouse brain showing the distribution of anterogradely labeled fibers following tracer injections in the MePV.** The injection site is depicted in panel **I**. **A** is rostral, **L** is caudal. The semi-schematic drawings were made based on two injections, one in a C57BL/J6 mouse and another in a CD1 mouse. For abbreviations, see list. Scale bar: 1 mm.

The labeled fibers resulting from the injections in the MePV course through the same pathways described above for the MeA and MePD injections, the *stria terminalis* and the ventral amygdalofudal pathway. In fact, a very dense group of axons leaving the injection site surrounds the MePD to reach the *stria terminalis* in their way out of the amygdala (Figure 8I). As reported with MePD efferents, no differences between strains appear in the anterograde labeling observed following tracer injections in the MePV. The output of the MePV is mostly ipsilateral, although a few axons appear in contralateral nuclei, as described below.

***Amygdala.*** Following MePV injections, dense anterograde labeling is found in the other subnuclei of the Me (Figures 8G–I). In the rest of the vomeronasal amygdala, there is very dense anterograde labeling in the PMCo (Figures 8I–L and 3C) and a dense labeling in the BAOT (not shown, see Table 1). In addition, the anterior amygdaloid area shows moderate labeling in its ventral part (Figure 8F). The AHi (which is strongly interconnected with the vomeronasal amygdala, Swanson and Petrovich, 1998) displays also moderately dense anterograde labeling (Figures 8I–K). In the olfactory amygdala, moderately dense axonal labeling is present in the ACo and the PLCo (Figures 8F–J), while the cortex-amygdala transition zone has only a very sparse anterograde labeling (Figures 8F,G). The LOT is mostly devoid of the MePV axons, but some fibers can be observed in layer 1 (Figure 8F). The central nucleus and associated intra-amygdaloid BST show scarce and heterogeneous anterograde labeling following the MePV injections, with light labeling in the CeM, only a few fibers in the CeL and no labeling in the CeC (Figures 8G–I). Scarce labeling is observed in the I and, in contrast, the BSTIA shows moderately dense labeling (Figures 8G–I). Finally, within the basolateral amygdaloid complex, only the basomedial nucleus has a moderate innervation both in its anterior and posterior parts (Figures 8G–J), whereas sparse anterograde labeling is observed in the ventral basolateral amygdaloid nucleus, and only a few fibers are present in the La (Figures 8G–J).

***Olfactory bulbs and cerebral cortex.*** Axons from the MePV course rostrally to innervate the AOB, where dense anterograde labeling is present in the granular layer and sparse labeling in the deep mitral cell layer (Figures 8A and 4B). In the olfactory system, sparse innervation is present in the Pir (with more labeled fibers present in its posterior zone) and dorsal *tenia tecta* (Figures 8B–K). In addition, very scarce labeling is present in the agranular insular cortex, endopiriform nucleus, and medial part of the anterior olfactory nucleus (Figures 8B–K). Within the hippocampal formation, a few axons extend caudally into the Ent and a moderate amount of labeling appears in the CA1 field of the hippocampus (Figure 8L).

***Septum, striatum, and bed nucleus of the stria terminalis.*** In the septal complex, at rostral levels the injections in the MePV result in anterograde labeling in the lateral septal nucleus (moderate in the LSV and scarce in the LSI and LSD divisions, Figures 8C,D). In addition, the SHy has a dense innervation (Figure 8D) while only a few axons are observed at the SHi (Figure 8C). Scarce labeling is found in the medial septum/diagonal band complex (Figures 8C,D).

In the ventral striatum, very scarce labeling is observed in the nucleus accumbens, Tu and islands of Calleja (Figures 8C,D). Also the SI has moderately dense labeling (Figures 8E–G) and very few fibers are present in the IPAC (Figure 8D).

From the injection site in the MePV labeled fibers run in the *stria terminalis* to reach the BST, where they give rise to a heterogeneous pattern of anterograde labeling. In the medial BST, labeling is very dense in the BSTMPM, dense in the BSTMA, intermediate in the BSTMPL and the BSTMPI, and relatively light and diffuse in the BSTMV (Figures 8D–F and 4F). In contrast, all the subnuclei of the lateral BST show very scarce anterograde labeling (Figures 8D,E; see Table 1).

***Hypothalamus.*** None of our injections in the MePV shows dense labeled terminal fields in the hypothalamus (Table 1). At **preoptic** levels, moderately dense labeling is observed in the MPO and sparse anterograde labeling appears in the MPA (Figures 8D–F), with very few fibers present in the LPO (Figures 8D,E). At **anterior** levels, the anterior hypothalamic area and LA show a low density of anterograde labeling (Figures 8F,G). In the **tuberal** region, there is a moderately dense labeling in the VMHVL and VMHDM (Figures 8G–I and 5C). Of note, dense labeling is observed just ventrolateral to the VMHVL (Figures 8H,I). A few labeled axons can be also observed in the LH and Arc, and very few in the DM (Figures 8F–J). At **mammillary** levels, the premammillary nucleus shows a few labeled fibers in the PMD, but a dense labeled terminal field in the PMV (Figures 8J and 5F). Also, a few fibers are observed in the SuM and MM (Figures 8K,L).

***Thalamus.*** The injections in the MePV result in anterograde labeling in a few thalamic nuclei. Some axons give rise to sparse terminal labeling in the PV and, to a lesser extent, in the Re and STh (Figures 8F–I). Axons progressing through the *stria medullaris* apparently provide a moderately dense innervation to the nucleus of the *stria medullaris* (Figure 8F).

***Brainstem and midbrain.*** Axons from the MePD course caudally and give rise to sparse anterograde labeling in the PAG and VTA (Figure 8L).

***Contralateral labeling.*** The anterograde labeling resulting from the injections in the MePV is mostly ipsilateral, but a few labeled axons cross the midline in the anterior commissure and the sox. In the contralateral hemisphere, anterograde labeling appears generally in those structures that present a dense ipsilateral projection. Thus, sparse anterograde labeling appears in the ventral lateral septum, BST, and the hypothalamus, mainly in MPO, anterior hypothalamic area, ventromedial hypothalamic nucleus, and PMV.

### Injections in the substantia innominata and the optic tract

In the injection located in the SI, dorsal to the MeA, we observe fiber labeling in the telencephalon coursing through the fmi, with anterograde labeling present in the parietal insular cortex and CPu. In the diencephalon, axonal labeling appears in the lateral LHb, lateral geniculate complex, ventral posterior thalamic nuclei, Po, STh, and sox. Finally, in the brainstem labeled fibers are located in the SN, both in its parts *compacta* and *reticulata*.

We obtained one injection restricted to the opt medial to the MePV. As expected, this case resulted in anterograde labeling being present in the different structures composing the visual thalamus, the pretectum and the superior colliculus (not shown).

## Discussion

The results presented in this work confirm in female mice the heterogeneity of the efferent projections of the Me previously reported in other male rodents (Gomez and Newman, 1992; Canteras et al., 1995; Coolen and Wood, 1998). This cytoarchitectonic and hodological compartmentalization of the Me is consistent also with the heterogeneous developmental territories that give rise to neuronal populations of this nucleus (García-López et al., 2008; García-Moreno et al., 2010; Bupesh et al., 2011), as well as with the expression of the genes encoding different transcription factors of the Lhx family (Choi et al., 2005).

In the present work we have compared the pattern of efferent projections of the anterior, posteroventral, and posterodorsal subdivisions of the Me in two different strains of mice, namely C57BL/6J (an inbred strain very often used in the generation of genetically modified animals) and CD1 (an outbred strain commonly used in behavioral studies). The results show that there are no relevant differences in the organization of the efferent projections of the three subdivisions between the two strains. In fact, the results we have found in female subjects of both strains are very similar to those reported in male rats and hamsters, suggesting that the inter-strain and inter-species differences in reproductive (Vale et al., 1973, 1974; Burns-Cusato et al., 2004; Dominguez-Salazar et al., 2004) and defensive (Belzung et al., 2001; Yang et al., 2004) behaviors cannot be attributed to differences in the pattern of efferent projections originated by the Me. Instead they may be due to differences at molecular level (e.g., expression of neurotransmitters or their receptors in the relevant connections). In addition, in the present work we have used female mice as experimental subjects. As far as we know, all of the previous reports of the efferent projections of the Me used male subjects (rats: Canteras et al., 1995; hamsters: Gomez and Newman, 1992; Coolen and Wood, 1998; mice: Choi et al., 2005; Usunoff et al., 2009). As already stated, the efferent projections of the Me subdivisions found in female mice are very similar to the previous results in male mice, as well as in male rats and hamsters. Since the medial amygdala, and specifically its posterodorsal subdivision, has been shown to be sexually dimorphic (Cooke et al., 1999), the present results suggest that the sexual dimorphism does not include the pattern of organization of its efferent projections (Simerly, 2002), at least as revealed by our qualitative study. Of course, quantitative analysis of particular pathways may uncover sexual differences in the magnitude of some of these efferent projections, or in their neurochemical features.

The efferent projections originated from all three subnuclei of the Me present a common component and a number of relevant differences. As described previously for the other amygdaloid efferents in the rat and mouse (Canteras et al., 1995; Petrovich et al., 1996; Novejarque et al., 2011) efferents from all three subnuclei of the Me course through the same tracts, namely the *stria terminalis* and the ventral amygdalofugal pathway (*ansa peduncularis*), which converge at rostral levels. Within the *stria terminalis*, the efferent projections from the Me course medially, as described also in male rats (Canteras et al., 1995) and male hamsters (Gomez and Newman, 1992). On the other hand, as reported in male rats (Canteras et al., 1995), the MeA contributes to the ventral amygdalofugal pathway more than the posterior Me. Experimental evidence in male hamsters suggests that the projection to the hypothalamus courses exclusively through the *stria terminalis* (Maragos et al., 1989). Consistent with this view, our results suggest that the ventral amygdalofugal pathway contains mainly fibers directed to the ventral striato-pallidum, some of which apparently continue rostrally to reach the AOB. In fact, the existence of a non-strial pathway from the vomeronasal amygdala to the AOB has been demonstrated in the mouse (Barber, 1982).

The projections of all three medial amygdaloid subnuclei are mainly ipsilateral, with only a few axons observed crossing the midline through the anterior commissure, the supraoptic commissure, and the supramammilary decussation. These contralateral axons lightly innervate the zones corresponding to the densest terminal fields observed in the ipsilateral hemisphere, as reported previously in male rats (Canteras et al., 1995).

The additional labeling that we obtained in the cases in which tracer leakage occurs along the pipette track does not interfere with the labeling found following restricted injections, since most of it terminates in different structures. The only target that probably receives a light projection from the Me and also an important one from the dorsally located *SI* is the LHb, as revealed by restricted injections in both MeA and SI (see “Results”). This is consistent with previous reports on the projections of the Me (Gomez and Newman, 1992; Canteras et al., 1995; Coolen and Wood, 1998) and of the SI (Grove, 1988). In addition, the SI also shows important projections to the VTA (Geisler and Zahm, 2005), and accordingly our injections in the Me and in the SI result in anterograde labeling in the VTA. Nevertheless, retrograde tracing after injections in the VTA confirm that at least the MeA and MePD do project to the VTA (Martinez-Hernandez et al., unpublished data).

### Intramygdaloid projections of the medial amygdaloid nucleus

As described in previous works, our results confirm in the mouse that the different subnuclei of the Me show dense bidirectional interconnections. However, our material reveals that the projections of the MePD to the rest of the Me are more important than suggested by previous studies in male rats (Canteras et al., 1995). By analysing the effects of lesions of the Me in the male Syrian hamster, Maras and Petrulis (2010a,b,c) proposed a functional interpretation for these anatomical data, which is fully supported by our findings in female mice. The MeA would filter the chemosensory information received from the olfactory bulbs that would then be relayed to the posterior medial amygdala. The abundant cells expressing receptors for sexual steroids in the MePD of the hamster (Wood et al., 1992) and the mouse (Mitra et al., 2003) make it a nodal center for the hormonal control of the response to odors and pheromones in the context of reproductive behavior. On the other hand, the projections from the MePD to the rest of the Me, which according to our results are very important in the mouse, would allow an integration of odor/pheromone information with endocrine signals.

In addition, it has been shown in male hamsters that the MeA responds to heterospecific chemosensory stimuli, whereas the MePD seems to be inhibited by this type of stimuli (Meredith and Westberry, 2004). It has been suggested that the anatomical basis of this phenomenon include a projection from the MeA to the GABA-enriched intercalated cell mass, which in turn would inhibit the MePD cells (Meredith and Westberry, 2004). Our anatomical results indicate that the projection from the MeA to the intercalated cell mass located next to the MePD is present in female mice, and therefore a similar mechanism may operate so that the reproductive-related circuit is inhibited by heterospecific odors. However, a recent study in rats show that the exposure to a live cat induce c-fos expression in all subdivisions of the medial amygdala (Martinez et al., 2011), thus suggesting that, at least in this situation, heterospecific-induced inhibition of the MePD does not take place.

The Me gives rise to important intramygdaloid projections, especially directed to the chemosensory amygdala (Gutiérrez-Castellanos et al., 2010). Among the projections to the olfactory amygdala (Kevetter and Winans, 1981a; Cadiz-Moretti et al., in press; see Figure 8), all three subdivisions project to the anterior cortical and posterolateral cortical nuclei, as it has been reported previously in different male rodents (hamsters, Coolen and Wood, 1998; rat, Canteras et al., 1995; mice, Usunoff et al., 2009). Our results reveal also a previously unnoticed sparse projection of the Me to the the cortico-amygdaloid transition area and the LOT (only mentioned by Gomez and Newman, 1992).

Among the projections innervating the vomeronasal amygdala (Kevetter and Winans, 1981b; see Figure 8), projections from the three subnuclei terminate in the posteromedial cortical nucleus and in the anterior amygdaloid area (which also receives direct projections from the AOB, Cadiz-Moretti et al., in press). In addition, the amygdalo-hippocampal transition area (which is not strictly chemosensory amygdala but it is strongly related to vomeronasal structures, see Swanson and Petrovich, 1998; Martínez-García et al., 2012), is also interconnected with the Me, especially with the posterodorsal and posteroventral divisions (present results, Canteras et al., 1992, 1995).

In summary, the Me not only receives direct projections from the main and AOBs (Scalia and Winans, 1975; Pro-Sistiaga et al., 2007; Kang et al., 2009, 2011; Cadiz-Moretti et al., in press), but also shows strong connections with both the olfactory and the vomeronasal components of the amygdala. In male rats and hamsters these intra-amygdaloid connections are reciprocal (Coolen and Wood, 1998; Pitkänen, 2000; Majak and Pitkänen, 2003), suggesting that the Me plays a relevant role in processing together the olfactory and vomeronasal chemical signals from conspecifics and heterospecifics (Baum and Kelliher, 2009; Keller et al., 2009; Martínez-García et al., 2009).

In contrast to the dense projection to the chemosensory cortical nuclei of the amygdala, the Me gives rise to relatively minor projections to the nuclei that compose the basolateral amygdaloid complex. Only the anterior basomedial nucleus apparently receives a moderate projection from all three medial amygdaloid subnuclei, although a minor projection to the lateral (ventral subnuclei) and basolateral nuclei also exists. These results are in agreement with the previous reports in several male rodents (Canteras et al., 1995; Coolen and Wood, 1998; Usunoff et al., 2009). Since the lateral and basolateral nuclei play a critical role in fear learning (Nader et al., 2001), the Me might play a secondary role in olfactory fear conditioning, an issue that remains to be clarified (Otto et al., 2000; Walker et al., 2005). Of note, when cat odor is used as unconditioned stimulus, the contextual fear acquired is dependent of both the basolateral and the medial amygdaloid nuclei (Li et al., 2004). In addition, the projection to the basolateral nucleus might also be involved in attaching incentive value to olfactory stimuli. In fact, the basolateral nucleus densely innervates the ventral striatum (Novejarque et al., 2011), a projection that is known to play a critical role in many motivated behaviors (Shiflett and Balleine, 2010; Stuber et al., 2011).

Finally, parts of the central nucleus of the amygdala receive substantial projections from the Me. In particular, the medial subdivision of the central nucleus receives moderate projections from all three Me subnuclei, and only the MeA gives rise to substantial projections to the capsular subdivision of the central nucleus. These results agree with previous reports in male mice (Usunoff et al., 2009) and hamsters (Gomez and Newman, 1992; Coolen and Wood, 1998), although in hamsters the capsular subdivision was not considered. The results reported in male rats are slightly different, since the MeA, MePD and MePV were found to moderately project to the capsular subdivision of the central nucleus and only the MeA gives rise to a moderate projection to the CeM (Canteras et al., 1995). The central nucleus of the amygdala is known to project to hypothalamic and brainstem targets that directly mediate fear and anxiety responses (Davis, 2000). These descending projections originate mainly from the medial division of the central nucleus (Rosen et al., 1991), and therefore the projections from the medial amygdala to the CeM are a direct link with a key structure mediating fear responses. However, lesions of the Me significantly reduce the unconditioned freezing induced by cat-derived odors, whereas lesions of the central nucleus have no effect (Li et al., 2004). Therefore, the functional significance of the projections from the Me to the CeM remains to be clarified.

### Projections of the medial amygdaloid nucleus to the bed nucleus of the stria terminalis, hippocampus, septum, and ventral striatum

The BST is a major recipient of efferent projections of the Me. Our results indicate that the different BST subdivisions display differential inputs from the subnuclei of the Me. Thus, the MeA gives rise to moderate projections to the lateral subdivisions of the BST (especially to the laterodorsal subnuclei of the BST). In contrast, the MePD and MePV originate only scarce projections to the lateral BST. This specific projection from the MeA to the lateral BST has not been reported in previous works (Gomez and Newman, 1992; Canteras et al., 1995), although a minor projection to lateral aspects of the anterior BST was illustrated by Coolen and Wood (1998). However, differences in the parcellation scheme of the BST may partially explain this discrepancy: in the rat projections from the MeA have been described to the anterolateral, subcommissural, and rhomboid subnuclei of the BST (Dong et al., 2001), which are included in the lateroventral BST and lateral posterior BST in our study (following the atlas of Paxinos and Franklin, 2004). In fact, a moderate input from the MeA to the lateroventral BST has been confirmed by means of retrograde tracing studies in the rat (Shin et al., 2008). In general, the lateral divisions of the BST are strongly interrelated with the central nucleus and are believed to be involved in eliciting fear and defensive or stress-related responses (Gray et al., 1993; Choi et al., 2007). A recent study in mice has revealed that the vomeronasal organ has many more receptors for predator-derived stimuli than it was previously believed (Isogai et al., 2011), and therefore it is not surprising that the Me has direct projections to the relevant areas of the BST. In this sense, the fact that mainly the MeA and, to a lesser extent, the MePV project to the lateral BST is consistent with the data showing that these subnuclei respond to predator-derived chemicals (Samuelsen and Meredith, 2009).

In contrast to the moderate and restricted projection to the lateral BST from the MeA, the medial subnuclei of the BST are densely innervated by all three medial amygdaloid subdivisions. The density of the main projections is different according to the subnucleus of origin: the MeA innervates most densely the medial posterointermediate BST subdivision, whereas the MePD innervates preferentially the medial posteromedial subdivision, and the MePV gives rise to the densest projection to the medial anterior BST. A similar pattern of projections has been shown previously for the MeA and MePD in several male rodents (hamsters: Gomez and Newman, 1992; Coolen and Wood, 1998; rats: Canteras et al., 1995; Dong et al., 2001; mice: Usunoff et al., 2009). It should be noted, however, that the efferents of the MePV have only been studied previously in male rats (Canteras et al., 1995; Dong et al., 2001). In our results the main projection from the MePV terminates in the medial anterior BST, whereas in male rats the main efferent from the MePV innervates the posterointermediate BST (interfascicular and transverse subnuclei of the posterior division, in their nomenclature, see Dong et al., 2001). Two alternative explanations to this discrepancy are possible, interspecies differences between rats and mice or sex differences between males and females. Further research on the MePV efferents will be necessary to solve this question.

The pattern of projections to the medial divisions of the BST is consistent with the proposed functional roles of the different subnuclei of the Me. The MeA seems to filter and categorize the received chemosensory information, which would be relayed: (1) to the neural circuit for socio-sexual behavior (pheromones) through projections to the MePD and posteromedial BST; and (2) to the neural circuit for defensive behavior (e.g., predator-derived chemosignals) through projections to the MePV and posterointermediate BST (Samuelsen and Meredith, 2009). In fact, the MePD projects massively to the posteromedial BST, and both nuclei are sexually dimorphic and enriched in steroid-sensitive cells involved in the control of sexual behavior (Mitra et al., 2003; Swann et al., 2009). In contrast, the MePV has been said to project mainly to structures involved in defensive behavior (Canteras, 2002; Choi et al., 2005). However, our results indicate that the MePV innervates the posteromedial BST and the anteromedial BST. The latter, in turn, gives rise to important projections to the hypothalamic neurosecretory system (Dong and Swanson, 2006), including the vasopressinergic and oxytocinergic neurons of the paraventricular nucleus. Since these neuropeptides in the Me have been shown to play a role in diverse social behaviors (Arakawa et al., 2010; Gabor et al., 2012), these data suggest that the MePV may be involved not only in defensive responses to predators, but also in the control of other non-sexual behaviors, such as agonistic encounters with same sex conspecifics or aversion to illness-derived social odors (Arakawa et al., 2010).

**Figure 9:**
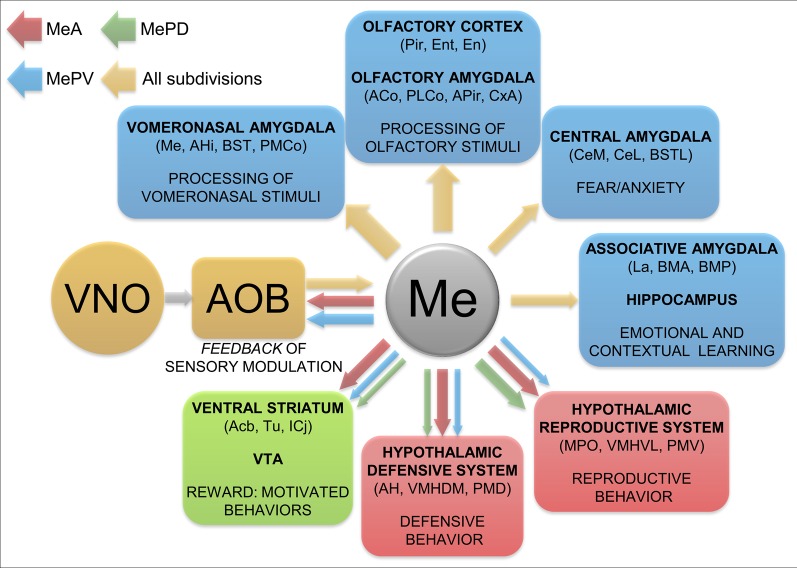
**Summary and functional interpretation of the efferent projections arisen from the medial amygdaloid nucleus.** Schematic representation of the main efferent projections of the medial amygdaloid nucleus, organized by functional systems. The differential projections of the different subdivisions are represented in red (MeA), green (MePD) and blue (MePV) colors. The thickness of the arrows roughly represents the density of the projections.

The three subdivisions of the Me give rise to moderate projections to the lateral septum, which target mainly its ventral and intermediate divisions. Noteworthy, the ventral division is enriched in vasopressin innervation, and this projection is sexually dimorphic (Wang et al., 1993) with more density of vasopressinergic terminals in males. This pathway may convey sociosexual (and maybe also predator-derived) chemosensory information to the lateral septum so that it can be integrated with the special and contextual information relayed by the hippocampal formation. The convergence of sociosexual and contextual information would allow the animal to elicit appropriate reproductive/defensive/aggressive behaviors as a function of its contextual situation (Lanuza and Martínez-García, 2009). In this regard, we have observed a minor projection to the ventralmost tip of the ventral hippocampus, which is common to all three subdivisions of the Me. Although this projection is certainly a very small one, it provides a direct route for the chemosensory stimuli processed in the Me to influence the contextual information processed in the ventral hippocampus (Figure 9). In rats, this same area of the ventral hippocampus has also been shown to receive substantial projections from the posteromedial cortical nucleus of the amygdala (Kemppainen et al., 2002) and to project back to AOB (de la Rosa-Prieto et al., 2009). Therefore, it is clearly a distinct subdivision of the ventral hippocampus that may be involved in integrating information about chemical signals with spatial or contextual cues.

According to our results, the Me displays scarce projections to some areas within the ventral striatum, mainly the ventromedial accumbens shell and adjacent Tu, which, as reported previously in male rats (Canteras et al., 1995) and hamsters (Gomez and Newman, 1992; Coolen and Wood, 1998), mainly arise from the MeA. The MePD and MePV contribute only with a few axons to this projection, although Usunoff et al. (2009) recently reported a significant projection from the MePD to the accumbens core, Tu and some islands of Calleja which we have not observed. The innervation in the medial Tu from the MeA reaches the vicinity of the ventromedial islands of Calleja. This area has also been reported to receive a direct input from the posteromedial cortical amygdala (Ubeda-Bañon et al., 2008; Novejarque et al., 2011), the other major target of the vomeronasal information. Therefore, this area of the ventral striato-pallidum may be specialized in processing the biological significance of pheromonal signals (Figure 9). In fact, the Tu is strongly related to the reward system of the brain (Ikemoto, 2007) and it may play a role in social odor processing (Wesson and Wilson, 2011).

### Differential centrifugal projections to the AOB from the medial amygdaloid subnuclei (Figure 9)

Our results confirm and extend previous works showing that the Me gives rise to important feedback projections to the AOB (Barber, 1982; Gomez and Newman, 1992; Canteras et al., 1995; Coolen and Wood, 1998; Fan and Luo, 2009). However, a number of differences between the present results and previous works should be highlighted. On the one hand, we have found that the projection from the MeA innervates mainly the ventral aspect of the mitral cell layer, in agreement with the description in mice (Barber, 1982), male rats (Canteras et al., 1995) and male hamsters (Gomez and Newman, 1992; Coolen and Wood, 1998). In the context of the cytoarchitecture of the AOB, this portion of the ill-organised mitral cell layer might represent the internal plexiform layer (Larriva-Sahd, 2008). However, a recent report in C57BL/6 male mice did not found this projection (Fan and Luo, 2009), maybe because the injections of retrograde tracers in the mitral cell layer of the AOB were too small. However, sex differences cannot be currently discarded. In addition, we also found that tracer injections in the MeA result in a few fibers in the glomerular layer of the posterior AOB, a light projection that may have important functional significance as it might modulate the input to particular glomeruli.

Regarding the feedback projection from the MePV to the granular layer of the AOB, it was not found in previous works in male rats (Canteras et al., 1995) or male hamsters (Gomez and Newman, 1992; Coolen and Wood, 1998), in the latter case probably because the injections in the posterior Me were located in a dorsal position. In contrast, this projection has been reported in male mice (Fan and Luo, 2009), and characterized as glutamatergic. Since the MePV contains a subpopulation of glutamatergic cells originated by the ventral pallium (Bupesh et al., 2011), it is likely that at least some of them give rise to the excitatory feedback projection to the granular layer of the AOB.

### Projections of the medial amygdaloid nucleus to the olfactory system

In addition to the projections targeting olfactory or vomeronasal amygdaloid nuclei already discussed, direct projections of the Me, originated mainly from the MeA, innervate a variety of structures of the olfactory system (including the piriform and entorhinal cortices, endopiriform nucleus, anterior olfactory nucleus and *tenia tecta*) (Figure 9). Only the MeA gives rise to significant projections to the anterior Pir, particularly around the lo, where they may converge with a light innervation directly originated by the mitral cells of the AOB (Cadiz-Moretti et al., in press). Therefore, as suggested previously (see Martínez-García et al., 2012), the Pir should not be viewed as a primary olfactory cortex, but as an associative chemosensory cortex.

### Projections of the medial amygdaloid nucleus to the hypothalamus

The hypothalamus is, together with the BST, the main target of the efferent projections of the Me. This fact has frequently led to the interpretation that the Me relays the information about pheromones, detected by the vomeronasal organ, to the hypothalamic circuits involved in the control of social and reproductive behavior (Tirindelli et al., 1998; Luo and Katz, 2004). However, as we have already discussed, the Me has a more complex role and, accordingly, its projections to the hypothalamus show a complex pattern (Choi et al., 2005). In general, the MeA and MePV project to hypothalamic structures involved both in reproductive and defensive behaviors, whereas the MePD shows a much more delimitated pattern of projections, innervating mainly hypothalamic structures known to be involved in reproductive behaviors (Petrovich et al., 2001; Choi et al., 2005). Our results are consistent with this pattern of organization (Figure 8), with some exceptions that are discussed below.

As described in male rats (Canteras et al., 1995; Petrovich et al., 2001) and hamsters (Gomez and Newman, 1992; Coolen and Wood, 1998), the hypothalamic targets of the MeA include not only the structures of the behavioral column controlling reproductive responses (Swanson, 2000), such as the MPO, ventrolateral VMH, and PMV, but also the structures of the behavioral column controlling defensive responses (Swanson, 2000; Canteras, 2002), such as the anterior hypothalamic area, dorsomedial VMH and (lightly innervated) the dorsal premmamillary nucleus. In contrast, the main hypothalamic targets of the MePD are the nuclei involved in reproductive behavior (MPO, anteroventral periventricular nucleus, ventrolateral VMH, and ventral premmamillary nucleus, Canteras et al., 1995; Petrovich et al., 2001). Regarding the hypothalamic projections of the MePV, our injections in this subdivision have resulted in relatively scarce anterograde labeling of hypothalamic structures. This may be due either to interspecific differences or to the presence of sexual dimorphism in these particular efferent projections. In any case, the major discrepancy of our results with those in male rats (Canteras et al., 1995; Petrovich et al., 2001) is the scarcity of projections to the hypothalamus of the MePV and, in particular, to the anterior hypothalamic area, which is part of the neural circuitry for defensive behaviors. In addition, we have found a very light projection to the dorsal premammillary nucleus, originated mainly in the MeA, but also in the MePD and MePV. A minor projection from the MeA and MePV to the dorsal premammillary nucleus has also been described in male rats using retrograde tracing (Comoli et al., 2000).

Another interesting result of our experiments is the presence of a relatively dense field of anterograde labeling in the PMV after tracer injections in the MePV. Although this confirms previous observations in the rat by Canteras et al. (1995), it does not fit a simple role of the MePV in anti-predatory defensive reactions, but suggests instead a possible modulation of socio-sexual behaviors mediated by this subnucleus. Since cells in the MePV are preferentially activated by predator-related chemosignals (Choi et al., 2005) this pathway might contribute to inhibiting sexual behavior in threatening contexts, an issue that clearly requires further study.

### Projections to the thalamus and the brainstem originated by the medial amygdaloid nucleus

The only thalamic nucleus that receives a dense input from the Me (all three subdivisions) is the nucleus of the *stria medullaris*, as described previously by Usunoff et al. (2009) in male mice. In addition, a light projection common to the three medial amygdaloid subdivisions innervate the paraventricular and reuniens nuclei of the midline thalamus. The Re projects (among other targets) to the piriform and entorhinal cortices (Vertes et al., 2006). Therefore, this thalamic relay provides an additional indirect pathway for vomeronasal information to influence olfactory inputs to the hippocampal formation. On the other hand, the paraventricular nucleus projects back to the Me, as well as to other amygdaloid structures and to the nucleus accumbens, Tu and bed nucleus of stria terminalis (Vertes and Hoover, 2008). These connections suggest that the paraventricular nucleus is likely involved in the set of emotional behaviors controlled by the amygdala/BST and the ventral striatum.

The Me provides only a light input to the brainstem, targeting the VTA and the PAG. The input to the VTA may be related to the reinforcing value that sexual pheromones have been shown to possess (Martínez-Ricós et al., 2007, 2008), although lesions of the dopaminergic cells of the VTA do not affect the attraction elicited by these sexual stimuli (Martínez-Hernández et al., 2006). In contrast, the projections of the PAG may be related to the elicitation of defensive behaviors by predator odors or chemical signals from dominant conspecifics (Motta et al., 2009). However, the few axons observed are not located in any of the longitudinal columnar regions of the periaqueductal grey described to be involved in different emotional behaviors (Bandler and Shipley, 1994), but oriented dorsoventrally next to the ventricle.

## Conclusions and functional remarks

The efferent projections of the three subdivisions of the Me revealed by our results show the following main characteristics (Figure 9):

1. The pattern of efferent projections of the medial amygdaloid subnuclei found in female mice is very similar to that reported in male rats and male hamsters. However, there are two relevant exceptions regarding the main projections originated from the MePV. First, in the present report in female mice we have found a very dense projection to the BSTMPM and a moderate projection to the BSTMPI, while the opposite pattern was found in male rats (Canteras et al., 1995). Second, in female mice the MePV gives rise to relatively light projections to the hypothalamus, whereas dense projections were reported in male rats (Canteras et al., 1995). Further research will be necessary to check whether these discrepancies are due to interspecific differences or to the presence of a certain degree of sexual dimorphism in these projections.
2. The Me (especially its anterior part) is strongly interconnected with the other structures receiving vomeronasal information from the AOB. This connectivity suggests that the information detected by the vomeronasal system is subjected to a complex intrinsic processing before being relayed to other structures. The necessity of complex processing of vomeronasal stimuli prior to its relay may be related not only to the complexity of the vomeronasal stimuli involving both volatile and involatile components (Zufall and Leinders-Zufall, 2007), but also to the fact that these vomeronasal stimuli are not merely related to reproductive behavior, as originally thought (Powers and Winans, 1975). For instance, it is known that the vomeronasal organ detects also aggression-eliciting chemical signals from same-sex conspecifics (Clancy et al., 1984; Chamero et al., 2007), molecules derived from a wide range of predators which induce defensive behaviors (Papes et al., 2010; Isogai et al., 2011), illness-related molecules (Rivière et al., 2009) which are likely to induce an avoidance response (Arakawa et al., 2010), and sulphated steroids present in the urine of conspecifics, which are likely to signal their physiological status (Nodari et al., 2008). Obviously, different neural circuits should be activated in response to each one of these vomeronasal stimuli.
3. The Me (especially its anterior part) has strong interconnections also with different structures of the main olfactory system, including the olfactory amygdala and the Pir. Therefore, there are anatomical pathways allowing for ample integration of olfactory and vomeronasal information in structures usually considered strictly as part of the olfactory system (Gutiérrez-Castellanos et al., 2010). This strongly supports new views on the complementary role of the olfactory and vomeronasal systems (Baum and Kelliher, 2009; Martínez-García et al., 2009).
4. The Me shows light projections to the basolateral amygdaloid complex and moderate projections to the central amygdala. These anatomical data suggest a minor role of the Me in fear learning (at least in the fear conditioning paradigm). In contrast, the Me has been showed to be involved in both unconditioned (Li et al., 2004) and learned (Takahashi et al., 2007) fear responses to predator odors.
5. The anterior and posteroventral subdivisions of the Me project to key structures of the circuit involved in the defensive response against predators. This circuit includes the posterointermediate part of the medial BST, the anterior hypothalamic area, the dorsomedial part of the VMH, and the dorsal premmamillary nucleus (Canteras, 2002). However, as suggested above, parts of this circuit may also be involved in agonistic behaviors, such as aggressive responses against same-sex conspecifics (Motta et al., 2009), or avoidance of parasitized conspecifics (Arakawa et al., 2010). Further research is needed to clarify whether there are subsystems within this global defensive system for these different behavioral responses. In this context, the projections of the MePV to nuclei related to reproductive behaviors (e.g., the ventral premammillary hypothalamus) can be interpreted as a pathway for inhibition of reproduction in the presence of predators or parasitized conspecifics.
6. The posterodorsal medial amygdaloid subnucleus mainly projects to the neural circuit for reproductive behaviors (Swanson, 2000), which includes the posteromedial part of the medial BST, the MPO, the ventrolateral part of the VMH, and the ventral premmamillary nucleus. Although there are numerous experimental evidences in support to this view (see Swann et al., 2009, for a review), we want to point out that parts of this circuit may be involved in non-sexual aspects of reproductive behavior, such as maternal aggression (Hasen and Gammie, 2006). In fact, a recent study has revealed the presence within the ventrolateral part of the VMH of male mice of mainly distinct neuronal subpopulations involved in fighting against male intruders and in mating (Lin et al., 2011). Therefore, the systems mediating reproductive and aggressive responses are not fully segregated.
7. It is interesting to note that the Me (especially the MeA) gives rise to light projections to the major structures of the brain system of reward, namely the VTA, the medial shell of the nucleus accumbens, and the Tu (including the islands of Calleja). Indirect projections to the ventral striatum also exist through the basolateral amygdala. Previous work has shown that male pheromones are attractive to female mice and can be used as appetitive unconditioned stimuli to induce learning (Moncho-Bogani et al., 2002; Agustín-Pavón et al., 2007; Martínez-Ricós et al., 2007; Ramm et al., 2008; Roberts et al., 2010). The projections from the Me to the reward system may be involved in processing the hedonic value of sexual pheromones. This hypothesis is consistent with data in hamsters showing that lesions of the Me abolish the female attraction toward male-derived chemical signals (Petrulis and Johnston, 1999). A recent report in mice has also shown that lesions of the medial amygdala blocked the preference of females for male urine, although in this case only lesions that included the posterior medial amygdala were effective (DiBenedictis et al., 2012), whereas lesions restricted to the anterior subdivision had no effect, suggesting that the connections originated by the posterior subdivisions are sufficient to mediate the female preference for male-derived chemicals. Of note, the basolateral amygdala is known to play a relevant role in reward learning (Baxter and Murray, 2002), but the extent to which the Me (and other structures of the chemosensory amygdala) participate in the rewarding aspects of pheromones (or chemical signals with hedonic value) is unknown.

### Conflict of interest statement

The authors declare that the research was conducted in the absence of any commercial or financial relationships that could be construed as a potential conflict of interest.

## Abbreviations

3V: 3rd ventricle
AAD: anterior amygdaloid area, dorsal part
AAV: anterior amygdaloid area, ventral part
ac: anterior commissure
AcbC: accumbens nucleus, core
AcbSh: accumbens nucleus, shell
ACo: anterior cortical amygdaloid nucleus
AHA: anterior hypothalamic area, anterior part
AHi: amygdalohippocampal area
AHP: anterior hypothalamic area, posterior part
AI: agranular insular cortex
AOB: accessory olfactory bulb
AOM: anterior olfactory nucleus, medial part
AON: anterior olfactory nucleus
AOP: anterior olfactory nucleus, posterior part
APir: amygdalopiriform transition area
Arc: arcuate hypothalamic nucleus
Astr: amygdalostriatal transition area
AVPe: anteroventral periventricular nucleus
BAOT: bed nucleus of the accessory olfactory
BLA: basolateral amygdaloid nucleus, anterior part
BLP: basolateral amygdaloid nucleus, posterior part
BLV: basolateral amygdaloid nucleus, ventral part
BMA: basomedial amygdaloid nucleus, anterior part
BMP: basomedial amygdaloid nucleus, posterior part
BST: bed nucleus of the *stria terminalis*
BSTIA: BST, intraamygdaloid division
BSTLD: BST, lateral division, dorsal part
BSTLI: BST, lateral division, intermediate part
BSTLP: BST, lateral division, posterior part
BSTLV: BST, lateral division, ventral part
BSTMA: BST, medial division, anterior part
BSTMPI: BST, medial division, posterointermediate part
BSTMPL: BST, medial division, posterolateral part
BSTMPM: BST, medial division, posteromedial part
BSTMV: BST, medial division, ventral part
CA1: field CA1 of hippocampus
cc: corpus callosum
CeC: central amygdaloid nucleus, capsular part
CeL: central amygdaloid nucleus, lateral division
CeM: central amygdaloid nucleus, medial division
Cl: claustrum
cp: cerebral peduncle
CPu: caudate putamen
CxA: cortex-amygdala transition zone
DEn: dorsal endopiriform nucleus
DM: dorsomedial hypothalamic nucleus
DTT: dorsal *tenia tecta*
ec: external capsule
Ent: entorhinal cortex
f: fornix
fmi: forceps minor of the corpus callosum
GlA: glomerular layer of the AOB
GrA: granule cell layer of the AOB
GrO: granular cell layer of the olfactory bulb
HDB: nucleus of the horizontal limb of the diagonal band
I: intercalated nuclei of the amygdala
ic: internal capsule
ICj: islands of Calleja
ICjM: major island of Calleja
IL: infralimbic cortex
IPAC: interstitial nucleus of the posterior limb of the anterior commissure
LA: lateroanterior hypothalamic nucleus
La: lateral amygdaloid nucleus
LH: lateral hypothalamic area
LHb: lateral habenular nucleus
lo: lateral olfactory tract
LOT: nucleus of the lateral olfactory tract
LPO: lateral preoptic area
LSD: lateral septal nucleus, dorsal part
LSI: lateral septal nucleus, intermediate part
LSV: lateral septal nucleus, ventral part
LV: lateral ventricle
MD: mediodorsal thalamic nucleus
Me: medial amygdaloid nucleus
MeA: medial amygdaloid nucleus, anterior part
MePD: medial amygdaloid nucleus, posterodorsal part
MePV: medial amygdaloid nucleus, posteroventral part
MGP: medial globus pallidus
MiA: mitral cell layer of the AOB
ML: medial mammillary nucleus, lateral part
MM: medial mammillary nucleus, medial part
MPA: medial preoptic area
MPO: medial preoptic nucleus
MS: medial septal nucleus
MTu: medial tuberal nucleus
opt: optic tract
Pa: paraventricular hypothalamic nucleus
PAG: periaqueductal gray
Pe: periventricular hypothalamic nucleus
PH: posterior hypothalamic area
Pir: piriform cortex
PLCo: posterolateral cortical amygdaloid nucleus
PMCo: posteromedial cortical amygdaloid nucleus
PMD: premammillary nucleus, dorsal part
PMV: premammillary nucleus, ventral part
Po: posterior thalamic nuclear group
PrL: prelimbic cortex
PSTh: parasubthalamic nucleus
PT: paratenial thalamic nucleus
PV: paraventricular thalamic nucleus
Re: reuniens thalamic nucleus
Rt: reticular thalamic nucleus
SCh: suprachiasmatic nucleus
SFi: septofimbrial nucleus
SHi: septohippocampal nucleus
SHy: septohypothalamic nucleus
SI: *substantia innominata*
SM: nucleus of the *stria medullaris*
sm: *stria medullaris*
SN: *substantia nigra*
sox: supraoptic decussation
st: *stria terminalis*
STh: subthalamic nucleus
SuM: supramammillary nucleus
TC: tuber cinereum area
Tu: olfactory tubercle
VDB: nucleus of the vertical limb of the diagonal band
VEn: ventral endopiriform nucleus
VMH: ventromedial hypothalamic nucleus
VMHC: ventromedial hypothalamic nucleus, central part
VMHDM: ventromedial hypothalamic nucleus, dorsomedial part
VMHVL: ventromedial hypothalamic nucleus, ventrolateral part
VP: ventral pallidum
VTA: ventral tegmental area
VTT: ventral *tenia tecta*
ZI: *zona incerta*.
